# Metabolic Flexibility and Inflexibility: Pathology Underlying Metabolism Dysfunction

**DOI:** 10.3390/jcm12134453

**Published:** 2023-07-03

**Authors:** Marni E. Shoemaker, Zachary M. Gillen, David H. Fukuda, Joel T. Cramer

**Affiliations:** 1 School of Health and Consumer Sciences, South Dakota State University, Brookings, SD 57007, USA; 2 Department of Kinesiology, Mississippi State University, 180 Magruder Street, Mississippi State, MS 39762, USA; zmg43@msstate.edu; 3 School of Kinesiology and Rehabilitation Sciences, University of Central Florida, Orlando, FL 32816, USA; david.fukuda@ucf.edu; 4 College of Health Professions and Sciences, University of Central Florida, Orlando, FL 32816, USA; joel.cramer@ucf.edu

Metabolic flexibility can be defined as the ability of the skeletal muscle to adjust its utilization of substrate pathways [1]. Timing this metabolic shift to available energy substrates is imperative to transition from the fasted to fed states and from rest to exercise states to meet energy needs. Thus, an inability to rapidly adjust energy substrate utilization has been termed metabolic inflexibility and has recently been associated with obesity, sarcopenia, insulin resistance, type 2 diabetes, and other metabolic chronic conditions [1,2,3]. Metabolic flexibility is typically measured in response to nutritional and exercise challenges with indirect calorimetry to determine energy expenditure from carbohydrates (CHO) and fat utilization [4]. These measurements, along with biomarkers of glucose and insulin, can provide a full picture of metabolic responses to nutritional and exercise stimuli in a variety of populations. Skeletal muscle is necessary for movement and exercise; however, it also has an important role in metabolism, as skeletal muscle accounts for 60–80% of the response of glucose to insulin [1,5]. Thus, to appropriately adjust substrate utilization in response to nutritional and/or exercise stress (i.e., glucose mobilization via insulin), increasing skeletal muscle quality and/or quality should be prioritized. Furthermore, substrate utilization and fuel adaptability are positively associated with exercise performance [6], while numerous dietary and training interventions have been explored to either quantify or manipulate these outcome variables [7,8,9,10]. These assessments can provide a better understanding of key factors related to metabolic flexibility, including physiological factors such as the energy demands of exercise.

The alignment of metabolic inflexibility and disease conditions related to metabolism seems to logically support using an “exercise as medicine” approach for disease improvement. For example, older adults with sarcopenia have lower skeletal muscle mass, strength, and function, and typically experience fat infiltration within skeletal muscle, leading to metabolic impairments that may foundationally contribute to the development of metabolic inflexibility. Recently, Shoemaker et al. examined differences in metabolic flexibility between sarcopenic and non-sarcopenic older adults. This study demonstrated that elderly adults with sarcopenia had greater CHO utilization at rest and post-prandial, and a diminished response to a CHO meal compared to non-sarcopenic elderly adults. Additionally, during aerobic exercise, fat oxidation was lower for the sarcopenic group than the non-sarcopenic group, suggesting an impairment in fat utilization and, thus, metabolic inflexibility, perhaps connected to mitochondrial dysfunction [2]. Conversely, in healthy children, Gillen et al. demonstrated that consumption of a rapid-digesting carbohydrate drink prior to exercise may promote greater exogenous carbohydrate utilization, demonstrating the ability of healthy children to adjust substrate utilization pathways based on dietary intake to maintain endogenous carbohydrate and fat sources, prolonging exercise during low-intensity bouts [11].

Both of these studies emphasize the importance of exercise for metabolic health across the lifespan, which is echoed in recent studies examining the role exercise has in metabolic-related chronic diseases [12,13,14,15]. A recent review highlighted the importance of resistance training for improving outcomes in the early and late stages of frailty and sarcopenia [15]. The conclusion of this review suggested that exercise may be a preventative strategy for preserving skeletal muscle health while highlighting its interrelatedness with chronic disease. Additionally, exercise training can promote metabolic responses, including improved fatty acid oxidation, insulin sensitivity, and mitochondrial content, which likely has corresponding benefits such as reducing insulin resistance, diabetes, cardiovascular disease, and other chronic conditions. For example, in females across the lifespan (ages 20–60 years), a combination of aerobic and resistance training improved risk factors of metabolic syndrome, including waist circumference, fasting blood glucose, blood pressure, and blood lipid parameters [12]. Additionally, Methenitis et al. examined differences in body composition, blood glucose, and lipid concentrations in sedentary compared to endurance-trained middle-aged adults consuming a high-fat diet [14]. This study found that training-induced energy expenditure is the main determinant of these metabolic parameters, as well as positive metabolic adaptations that were more influenced by exercise training rather than nutritional intake. The authors theorized that the training volume resulted in improved mitochondrial density and oxidative capacity, leading to improved regulation of CHO and fat metabolism, thus making endurance-trained individuals more metabolically flexible than sedentary individuals [14]. Similarly, a study assessing functional fitness abilities in individuals with and without metabolic syndrome reported that the fitness level was lower in those with metabolic syndrome, independent of sex and age [13], and individuals with type 2 diabetes and coronary artery disease had low aerobic exercise capacity [16], further supporting the idea that exercise may be an important driver of improving metabolic flexibility.

Across the lifespan, it seems that improvements in metabolic flexibility may also be driven by improvements in lean body mass [17,18]. Oh et al. examined the association of changes in predicted body composition and metabolic health with metabolic syndrome, finding that greater muscle mass reduced the risk of metabolic syndrome in both males and females [17]. Furthermore, it has been demonstrated that adults with low skeletal muscle mass and high adiposity are at greater risk for high glucose, triglycerides, and other biomarkers associated with metabolic health [18]. Since exercise can have a profound effect on increasing lean body mass, even independent of nutritional interventions, it stands to reason that exercise may be an important component of improving metabolic flexibility.

In conclusion, since metabolic flexibility can provide unique insight into metabolic and overall health, it may be pertinent to prioritize specific interventions to affect this important health-related outcome. Based on the current literature, exercise has the potential to act as a “medicine” that may improve metabolic flexibility. In conjunction with appropriate nutritional recommendations, an emphasis on improving muscle quality and quantity through a well-balanced exercise regimen may help individuals improve metabolic flexibility, thus reducing the potential for metabolic diseases and disorders.
